# IGF-1, IGF-2 and IGFBP-3 in prediagnostic serum: association with colorectal cancer in a cohort of Chinese men in Shanghai

**DOI:** 10.1054/bjoc.2001.2172

**Published:** 2001-11

**Authors:** N M Probst-Hensch, J-M Yuan, F Z Stanczyk, Y-T Gao, R K Ross, M C Yu

**Affiliations:** Institute of Social and Preventive Medicine, University of Basel, Steinengraben 49, Basel, 4051, Switzerland; USC/Norris Comprehensive Cancer Center, Keck School of Medicine, University of Southern California, Los Angeles, CA, 90033, USA; Dept. of OB/GYN, Keck School of Medicine, University of Southern California, Los Angeles, CA, 90033, USA; , Shanghai Cancer Institute, Shanghai, 200032, Peoples' Republic of China

**Keywords:** IGF-1, IGF-2, IGFBP-3, colorectal cancer

## Abstract

This is the first study to investigate the associations of IGF-1, IGF-2 and IGFBP-3 concentrations with the risk of colorectal cancer in prospectively collected blood samples from an Oriental population. Between 1986 and 1989 serum samples were collected at baseline from 18 244 men, aged 45–65 years, without a history of cancer and living in Shanghai, China. IGF-1, IGF-2 and IGFBP-3 were measured in the serum of 135 men who developed colorectal cancer over 12 years of follow-up and 661 control subjects drawn from the cohort, who were matched to the index cases by neighbourhood of residence, age, and year and month of sample collection. Serum IGF-1 was not associated with risk of colorectal cancer. IGF-2 and IGFBP-3, on the other hand, exhibited statistically significant, positive associations with colorectal cancer risk when cases were confined to those diagnosed within a relatively short time period after enrolment (within 8 years). After adjustment for body mass index, cigarette smoking and alcohol intake, men in the highest versus the lowest quintile of IGF-2 and IGFBP-3 showed odds ratios of 2.74 (95% Cl = 1.67–4.50; 2-sided *P* for trend = 0.0008) and 2.85 (95% Cl = 1.69–4.81; 2-sided *P* for trend = 0.01), respectively. Our data thus suggest that circulating IGF-2 and IGFBP-3 can serve as early indicators of impending colorectal cancer. © 2001 Cancer Research Campaign http://www.bjcancer.com


*British Journal of Cancer* (2001) **85**(11), 1695–1699
© 2001 Cancer Research Campaign
doi: 10.1054/ bjoc.2001.2172, available online at http://www.idealibrary.com on IDEAL

http://www.bjcancer.com

### IGF-1, IGF-2 and IGFBP-3 in prediagnostic serum: association with colorectal cancer in a cohort of Chinese men in Shanghai

**NM Probst-Hensch[1], J-M Yuan[3], FZ Stanczyk[3], Y-T Gao[4], RK Ross[2] and MC Yu[2]**

[1]Institute of Social and Preventive Medicine, University of Basel, Steinengraben 49, 4051 Basel, Switzerland; [2]USC/Norris Comprehensive Cancer Center, Keck School of Medicine, University of Southern California, Los Angeles, CA 90033, USA; [3]Dept. of OB/GYN, Keck School of Medicine, University of Southern California, Los Angeles, CA 90033, USA; [4]Shanghai Cancer Institute, Shanghai 200032, Peoples' Republic of China

**Summary** This is the first study to investigate the associations of IGF-1, IGF-2 and IGFBP-3 concentrations with the risk of colorectal cancer in prospectively collected blood samples from an Oriental population. Between 1986 and 1989 serum samples were collected at baseline from 18 244 men, aged 45–65 years, without a history of cancer and living in Shanghai, China. IGF-1, IGF-2 and IGFBP-3 were measured in the serum of 135 men who developed colorectal cancer over 12 years of follow-up and 661 control subjects drawn from the cohort, who were matched to the index cases by neighbourhood of residence, age, and year and month of sample collection. Serum IGF-1 was not associated with risk of colorectal cancer. IGF-2 and IGFBP-3, on the other hand, exhibited statistically significant, positive associations with colorectal cancer risk when cases were confined to those diagnosed within a relatively short time period after enrolment (within 8 years). After adjustment for body mass index, cigarette smoking and alcohol intake, men in the highest versus the lowest quintile of IGF-2 and IGFBP-3 showed odds ratios of 2.74 (95% CI = 1.67–4.50; 2-sided *P* for trend = 0.0008) and 2.85 (95% CI = 1.69–4.81; 2-sided *P* for trend = 0.01), respectively. Our data thus suggest that circulating IGF-2 and IGFBP-3 can serve as early indicators of impending colorectal cancer. © 2001 Cancer Research Campaign http://www.bjcancer.com

**Keywords**: IGF-1; IGF-2; IGFBP-3; colorectal cancer

Insulin-like growth factors-1 and -2 (IGF-1 and IGF-2) are polypeptide growth factors which play a crucial role in cell cycle progression and cell proliferation as well as in cell differentiation and cell death. They exert their mitogenic, cell transforming and antiapoptotic signals by binding with high affinity to the IGF-1-receptor (IGF-1R). The autocrine/paracrine and endocrine interaction of IGFs with the IGF-1R is modulated by one of at least 6 insulin-like growth factor-binding proteins (IGFBPs). The IGFBPs bind IGF-1 and IGF-2 with affinity constants that are between 2- and 50-fold greater than those for IGF-1R. In the blood, the principal carrier of IGF-1 and IGF-2 is IGFBP-3. Because of its high affinity constant, which is 10- to 20-fold greater than either IGFBP-1 or -2, the other major forms of IGFBPs in the blood, IGFBP-3 binds 90–96% of IGF-1 and IGF-2 (Jones and Clemmons, 1995).

Experimental and epidemiological evidence have suggested an important role of the growth hormone (GH)-IGF-axis in the aetiology and pathophysiology of colorectal tumours (Freier et al, 1999; Giovannucci, 1999). In this report, we present results from the first study to investigate the associations between colorectal cancer risk and concentrations of circulating IGF-1, IGF-2 and IGFBP-3 in Chinese men using prospectively collected blood samples.

*Received 12 June 2001*
*Revised 5 September 2001*
*Accepted 10 September 2001*

*Correspondence to:* N Probst-Hensch

### MATERIALS AND METHODS

### Subjects

Subjects were drawn from a prospective study of men in Shanghai, China (the Shanghai Cohort Study). Details of the cohort have been previously published (Yuan et al, 1996). In brief, between January 1, 1986 and September 30, 1989, all male residents of 4 small geographically defined communities in Shanghai, who were aged 45–64 years and had no history of cancer, were invited to participate in an epidemiological study. At enrolment, subjects provided a 10 ml blood sample and a single void of urine. Samples of serum were stored in both –70˚C and –20˚C freezers. Serum samples used in the present study were those stored in -20˚C freezers. Each subject was interviewed in person using a structured questionnaire that included demographic information, history of tobacco and alcohol use, current diet (45 items) and medical history. A total of 18 244 men were enroled, constituting approximately 80% of eligible subjects. Follow-up has been conducted by annual contacts with all surviving cohort members and twice yearly review of cancer reports from the population-based Shanghai Cancer Registry and of death certificates. By September 1998, 207 subjects had been lost to follow-up. Written informed consent was obtained from all subjects in the study and the described investigations were approved by both the University of Southern California and the Shanghai Cancer Institute Institutional Review Boards.

Through follow-up ending September 1998, we identified 141 incident cases of colorectal cancer. For each case of incident colorectal cancer, 5 controls were drawn with matching on neighbourhood of residence, age at interview (within 2 years) and year and month of sample collection. Statistical analysis was restricted to subjects with complete measurements of all 3 IGF-components. The sample size for this study, therefore, is 135 cases and 661 control subjects, corresponding to 135 matched sets. The median age at diagnosis among cases was 58 years. There were 66 colon cancer and 69 rectal cancer cases. 129 cases were histopathologically confirmed. Among cases, the time interval between recruitment and cancer diagnosis ranged from 3 months to 12.3 years, with a mean of 6.1 years.

### Laboratory analysis

Serum samples, including some quality control samples, were sent to the laboratory of FZ Stanczyk for measurement of IGF-1, IGF-2 and IGFBP-3. Laboratory personnel were blinded to case–control or quality control status of the samples. IGF-1 was quantified by radioimmunoassay using commercial kits from Quest Diagnostics at Nichols Institute (San Juan Capistrano, California). IGF-2 and IGFBP-3 were measured by immunoradiometric assays with kits obtained from Diagnostic Systems Laboratory (Webster, Texas). Samples pertaining to matched subjects were assayed within a single assay run (batch). 2 duplicate aliquots of quality control samples were inserted in each of 24 batches to assess within-batch variation. While IGF-2 and IGFBP-3 measurements were obtained for all 48 quality control samples, IGF-1 values were only available for 45 quality control samples. The mean intra-batch coefficients of variation (standard deviation (SD)) for IGF-1, IGF-2 and IGFBP-3 from the duplicate quality control samples were 5.4% (SD 5.3), 3.2% (SD 4.0) and 6.5% (SD 6.8), respectively. Complete IGF-1, IGF-2 and IGFBP-3 measurements were performed on 135 case subjects and on 661 controls subjects, respectively.

### Statistical analysis

Data were analysed using SAS version 6.12 (Cary, NC). The conditional logistic regression method was used to examine associations between serum IGFs and risk of colorectal cancer (Hosmer and Lemeshow, 1989). The magnitude of the associations were measured by odds ratios (ORs) and their corresponding 95% confidence intervals (Cls) and *P* values. All *P* values quoted are 2-sided. Quintiles for IGF components were formed based on the distributions of the measurements among control subjects. The linear trend tests for exposure-disease associations were based on continuous variables. In 5 of the 24 batches, one of the 2 duplicate quality control samples demonstrated visibly larger variability than the remaining 43 pairs of quality control samples with regard to either IGF-1, IGF-2 or IGFBP-3 measurements. Statistical analysis of data excluding either these 5 batches or the batch with no IGF-1 quality control measurements did not materially alter the findings of this study. All results presented are, therefore, based on the total 135 cases and 661 control subjects.

### RESULTS

Among control subjects, IGF-1, IGF-2 and IGFBP-3 were highly correlated (IGF-1 vs. IGF-2: Spearman r = 0.51; IGF-2 vs. IGFBP-3: r = 0.53; IGF-2 vs. IGFBP-3: r = 0.56; $P \leq 0.0001$ for all r's). There were modest, but statistically significant correlations between the IGF components and selected demographic factors, specifically, between IGF-1 and age (r = –0.19, *P* = 0.0001), height (r = 0.12, *P* = 0.003), weight (r = 0.19, *P* = 0.0001), body mass index (BMI; weight/height squared) (r = 0.16, *P* = 0.0001), alcohol intake (g of ethanol per day) at baseline (r = 0.12, *P* = 0.003), and cigarette consumption (number of cigarettes smoked per day) at baseline (r = –0.12, *P* = 0.002); between IGF-2 and age (r = –0.11, *P* = 0.005), height (r = 0.11, *P* = 0.007), and weight (r = 0.09, *P* = 0.03); and between IGFBP-3 and height (r = 0.10, *P* = 0.01), weight (r = 0.16, *P* = 0.0001), and BMI (r = 0.12, *P* = 0.002).

No statistically significant differences between case and control subjects were reported with regard to anthropometric variables. Mean height, weight and BMI for cases and controls were (168.6, 168.0 cm), (64.0, 63.0 kg), and (22.5, 22.3 kg $m^{-2}$), respectively. Alcohol and cigarette consumption were comparable between cases and controls (weekly alcohol intake at baseline: 40% and 42%, respectively; daily cigarette consumption at baseline: 52% vs 49%, respectively).

Table 1 shows associations of IGF-1, IGF-2 and IGFBP-3 with risk of colorectal cancer after adjustment for cigarette consumption, alcohol intake and BMI. IGF-1 exhibited a statistically non-significant positive association with colorectal cancer risk, which disappeared after adjustment for IGFBP-3. For IGF-2, a 2.2-fold elevated risk was observed for men in the highest versus the lowest quintiles (*P* for trend = 0.03). The effect was diminished after adjustment for IGFBP-3 (OR = 2.04, *P* for trend = 0.14). Similarly, the positive association between IGFBP-3 and colorectal cancer risk (*P* for trend = 0.07) was diminished when adjustments were made for IGF-1 (OR = 1.78, *P* for trend = 0.13) or IGF-2 (OR = 1.31, *P* for trend = 0.60). None of the 3 molar ratios, IGF-1/IGFBP-3, IGF-2/IGFBP-3 and (IGF-1 + IGF-2)/IGFBP-3, differed significantly between cases and controls (Table 1).

The potential modification of the observed associations by duration of follow-up was investigated (Table 2). Due to sample size restrictions, quintiles for the IGF-components were collapsed to form 2 levels only. Grouping of quintiles was based on quintile/risk associations as presented in Table 1. There was no statistically significant association between IGF-1 and colorectal cancer risk, irrespective of duration of follow-up. Both the IGF-2 and IGFBP-3 associations with risk of colorectal cancer varied with the duration of follow-up. For both IGF-2 and IGFBP-3, no association with colorectal cancer was observed beyond 8 years of follow-up. IGF-2 showed a statistically significant association with colorectal cancer within the first 8 years of follow-up (OR = 3.53 for the first 4 years of follow-up, and 2.41 for years 4–8 of follow-up). These associations were slightly diminished after adjustment for IGFBP-3, but remained statistically significant. Similarly, IGFBP-3 was positively and statistically significantly associated with colorectal cancer within the first 8 years of follow-up (OR = 2.83 for the first 4 years of follow-up, and 2.85 for years 4–8 of follow-up). Adjustment for either IGF-1 or IGF-2 had only minor effects on the positive IGFBP-3/colorectal cancer risk associations. Irrespective of duration of follow-up no association between cancer risk and the molar ratio (IGF-2/IGFBP-3) was observed.

No substantial modification of either the IGF-1 or the IGFBP-3 effect by cancer site was observed (data not shown). A strong association was noted between rectal cancer and IGF-2 concentrations within the first 4 years of follow-up without and with IGFBP-3 adjustment. The ORs (95% Cls) were 7.00 (2.10–23.27) and 6.51 (1.62–26.14), respectively.

 *© 2001 Cancer Research Campaign*

**Table 1** Odds ratios (OR)[a] of colorectal cancer according to quintiles of prediagnostic serum levels of IGF-1, IGF-2 and IGFBP-3

| | Quintile | | | | | ***P* for trend** |
|---|---|---|---|---|---|---|
| | **1 (referent)** | **2** | **3** | **4** | **5** | |
| IGF-1 | | | | | | |
| No. case/controls | 24/135 | 25/135 | 27/130 | 25/130 | 34/131 | |
| Mean, ng ml$^{-1}$ | 100 | 135 | 159 | 188 | 246 | |
| OR | 1.00 | 1.04 | 1.20 | 1.12 | 1.52 | 0.34 |
| 95% CI | – | 0.56–1.93 | 0.65–2.21 | 0.59–2.11 | 0.82–2.85 | |
| OR, IGFBP-3-adjusted[b] | 1.00 | 0.97 | 1.05 | 0.96 | 1.18 | 0.96 |
| 95% CI | – | 0.51–1.82 | 0.55–2.01 | 0.48–1.91 | 0.55–2.53 | |
| IGF-2 | | | | | | |
| No. case/controls | 22/136 | 22/133 | 21/132 | 31/129 | 39/131 | |
| Mean, ng ml$^{-1}$ | 458 | 579 | 653 | 738 | 901 | |
| OR | 1.00 | 1.05 | 1.03 | 1.61 | 2.20 | 0.03 |
| 95% CI | – | 0.55–2.01 | 0.53–2.00 | 0.86–3.02 | 1.15–4.20 | |
| OR, IGFBP-3-adjusted[b] | 1.00 | 1.03 | 0.98 | 1.52 | 2.04 | 0.14 |
| 95% CI | – | 0.53–1.98 | 0.49–1.99 | 0.76–3.04 | 0.96–4.33 | |
| IGFBP-3 | | | | | | |
| No. case/controls | 21/134 | 15/133 | 36/128 | 30/131 | 33/135 | |
| Mean, ng ml$^{-1}$ | 1432 | 1824 | 2096 | 2385 | 3006 | |
| OR | 1.00 | 0.73 | 1.93 | 1.58 | 1.72 | 0.07 |
| 95% CI | – | 0.36–1.49 | 1.05–3.53 | 0.84–2.98 | 0.91–3.25 | |
| OR, IGF-1 adjusted[b] | 1.00 | 0.74 | 1.96 | 1.62 | 1.78 | 0.13 |
| 95% CI | – | 0.36–1.52 | 1.04–3.70 | 0.82–3.23 | 0.86–3.70 | |
| OR, IGF-2 adjusted[b] | 1.00 | 0.67 | 1.68 | 1.29 | 1.31 | 0.60 |
| 95% CI | – | 0.33–1.39 | 0.87–3.22 | 0.62–2.67 | 0.59–2.91 | |
| Molar ratio (IGF-1/IGFBP-3) | | | | | | |
| No. case/controls | 28/130 | 27/132 | 25/133 | 27/134 | 28/132 | |
| Mean, ng ml$^{-1}$ | 0.182 | 0.235 | 0.273 | 0.317 | 0.403 | |
| OR | 1.00 | 0.93 | 0.86 | 0.91 | 0.96 | 0.52 |
| 95% CI | – | 0.51–1.68 | 0.46–1.59 | 0.49–1.72 | 0.48–1.89 | |
| Molar ratio (IGF-2/IGFBP-3) | | | | | | |
| No. case/controls | 25/133 | 29/131 | 27/132 | 31/130 | 23/135 | |
| Mean, ng ml$^{-1}$ | 0.836 | 1.044 | 1.165 | 1.299 | 1.605 | |
| OR | 1.00 | 1.16 | 1.09 | 1.23 | 0.81 | 0.37 |
| 95% CI | – | 0.63–2.12 | 0.58–2.03 | 0.65–2.31 | 0.38–1.72 | |
| Molar ratio (IGF-1 + IGF-2/IGFBP-3) | | | | | | |
| No. case/controls | 23/136 | 27/132 | 30/129 | 32/126 | 23/138 | |
| Mean, ng ml$^{-1}$ | 1.069 | 1.303 | 1.443 | 1.594 | 1.947 | |
| OR | 1.00 | 1.25 | 1.41 | 1.51 | 0.91 | 0.33 |
| 95% CI | – | 0.67–2.35 | 0.76–2.62 | 0.79–2.88 | 0.42–1.97 | |

[a]all ORs are adjusted for cigarette smoking (number of cigarettes/day), alcohol intake (g ethanol/day), and body mass index (actual values) at baseline.
[b]OR additionally adjusted for other IGF-components as stated.

**Table 2** Odds ratios (OR)[a] of colorectal cancer according to IGF-2 and IGFBP-3 levels, by duration of follow-up

| | Duration of follow-up, years (mean) | | | | | |
|---|---|---|---|---|---|---|
| | **< 4 (2.3)** | | **4–8 (6.0)** | | **≥ 8 (9.9)** | |
| | **Ca/Co** | **OR (95% CI)** | **Ca/Co** | **OR (95% CI)** | **Ca/Co** | **OR (95% CI)** |
| IGF-2 | | | | | | |
| 1st–3rd quintiles | 13/106 | 1.0 | 29/200 | 1.0 | 23/95 | 1.0 |
| 4th–5th quintiles | 21/59 | 3.53 (1.48–8.44)a | 39/132 | 2.41 (1.31–4.41)a | 10/69 | 0.61 (0.25–1.48)a |
| | | 2.92 (1.03–8.28)b | | 2.25 (1.16–4.37)b | | 0.66 (0.25–1.74)b |
| IGFBP-3 | | | | | | |
| 1st–2nd quintiles | 8/75 | 1.0 | 15/140 | 1.0 | 13/52 | 1.0 |
| 3th–5th quintiles | 26/90 | 2.83 (1.18–6.79)a | 53/192 | 2.85 (1.48–5.48)a | 20/112 | 0.73 (0.31–1.71)a |
| | | 2.49 (0.96–6.43)c | | 3.20 (1.51–6.78)c | | 0.77 (0.30–1.95)c |
| | | 1.80 (0.67–4.85)d | | 2.56 (1.21–5.41)d | | 0.95 (0.36–2.49)d |
| Molar Ratio (IGF-2/IGFBP-3) | | | | | | |
| 1st–3rd quintiles | 20/91 | 1.0 | 38/199 | 1.0 | 23/106 | 1.0 |
| 4th–5th quintiles | 14/74 | 0.83 (0.34–2.07)[a] | 30/133 | 1.32 (0.72–2.44)[a] | 10/58 | 0.62 (0.22–1.74)[a] |

[a]ORs adjusted for body mass index (actual values), alcohol intake (g ethanol/day) and cigarette consumption (number of cigarettes/day) at baseline.
[b]ORs were additionally adjusted for IGFBP-3.
[c]ORs were additionally adjusted for IGF-1.
[d]ORS were additionally adjusted for IGF-2.

*© 2001 Cancer Research Campaign*

### DISCUSSION

In the present study conducted in an Oriental population we found that colorectal cancer cases had elevated circulating levels of IGF-2 and IGFBP-3, which were evident only relatively shortly (i.e., within 8 years) before their clinical diagnosis of cancer. Previous case–control and prospective studies involving mostly Caucasian subjects have reported a rather consistent, though not always statistically significant, positive association between circulating IGF-1 levels and colorectal cancer. The epidemiological data are equivocal with regard to the association of colorectal cancer and endocrine IGFBP-3 or IGF-2 levels (El-Atiq et al, 1994; Ma et al, 1999; Manousos et al, 1999; Giovannucci et al, 2000; Kaaks et al, 2000). The confinement of the IGF-2 and IGFBP-3 effects to cases diagnosed within 8 years of enrolment in our study is a novel finding.

The elevation in circulating IGF-components in the 8 years preceding cancer diagnosis is unlikely to reflect behavourial changes associated with early cancer symptoms, since acute fasting as well as chronic caloric and protein restriction lead to either no change or a decrease in circulating IGF-1, IGF-2 and IGFBP-3 concentrations (El-Atiq et al, 1994; Thissen et al, 1994).

Our results are compatible with a role of IGF-2 and IGFBP-3 elevations in the multistep process of colorectal carcinogenesis. In vitro evidence indicates that IGF-2 stimulates growth of human colorectal cancer and probably normal epithelial cells via an autocrine pathway. Tumours which proliferate in response to autocrine growth factors appear to grow more rapidly (Lamonerie et al, 1995a). Neoplastic conversion of colonic cells is associated with overexpression of IGF-2 gene and protein (Freier et al, 1999). A high level of amplification and expression of the c-myc gene, which is accompanied by an overexpression of IGF-2, differentiates tumorigenic from non-tumorigenic clones of SW613-S human colon carcinoma cell lines in a nude mice model. Proliferation of the tumorigenic clones in vitro is dependent on the autocrine production of IGF-2 (Lamonerie et al, 1995a, 1995b). The mechanisms underlying the IGF-2 overexpression and the associated increase in proliferation and tumorigenicity of colorectal cancer cell lines are subject to ongoing research. Activating mutations of the ras gene family and constitutive activation of $pp60^{c\text{-}src}$, the product of the src proto-oncogene, are among the genetic and epigenetic alterations most frequently observed in human colorectal cancer. In CaCo-2 cell lines which exhibit these frequent alterations, the 2 oncogenes deregulate the proliferation of human colonic cells by stimulating IGF-II production through a protein kinase C pathway. This pathway presumably stimulates interaction of the transcription factor Sp1 with several specific binding sites in P3 and P4 promoters of the IGF-2 gene (Cadoret et al, 1998). Studies of CaCo-2 colon carcinoma cell lines suggest that IGF-2 overexpression then impacts on cell proliferation by up-regulating the expression of cyclo-oxygenase-2 and prostaglandin E2 (Popolo et al, 2000).

The in vitro results are compatible with results from animal studies. Genetic manipulation to increase IGF-2 expression in the intestinal tract of 24 ApcMin/+ mice, a murine model of human familial adenomatous polyposis, increased the number and diameter of colon adenomas and the likelihood of histological progression to carcinoma in comparison to 17 ApcMin/+ mice with normal IGF-2 expression (Hassan and Howell, 2000). Growth hormone-releasing hormone (GH-RH) antagonists were found to inhibit growth of human HT-29 colon cancer cells transplanted into male nude mice. The treatment, which was started between 7 and 19 days after the transplantation of colon cancer cells, significantly decreased protein and expression levels of IGF-2 in the developing cancers when compared to control mice not treated with GH-RH antagonists (Szepeshazi et al, 2000).

Thus, experimental evidence is compatible with a central role of IGF-2 in neoplastic transformation and progression of colon epithelial cells. Interestingly, this possible aetiologic role of IGF-2 may be reflected in its peripheral blood levels. Among volunteers aged 55–64 undergoing flexible sigmoidoscopy, IGF-2 protein was overexpressed in adenomatous but not hyperplastic polyps. Circulating IGF-2 levels, which also were high in subjects with adenomatous polyps, dropped to normal levels after polyp removal (Renehan et al, 2000). Circulating IGF-2 levels, thus, may serve as a risk marker in individuals with colorectal adenomas, a hypothesis that is compatible with our study results. A role of IGF-2 in cancer susceptibility is also suggested by loss of imprinting (LOI) of the IGF-2 gene in both tumour tissue and matched normal colonic mucosa of 44% of colorectal cancer patients (Cui et al, 1998).

There is limited laboratory evidence in support of a positive association between IGFBP-3 and colorectal tumour progression. Experiments with colon cancer cell lines indicate that IGFBP-3, bound to cell surfaces or to the extracellular matrix, potentiates IGF-1 action by enhancing its interaction with the IGF-1R (Jones and Clemmons, 1995; Ferry et al, 1999). Upon exposure to transforming growth factor (TGF) beta 1, many human colon carcinoma cells undergo proliferation and exhibit a highly metastatic phenotype. In vitro results suggest that the proliferative TGF beta 1 effect may be mediated through the induction of IGFBP-3 (Kansra et al, 2000). The relevance of circulating IGFBP-3 on the biological activity of IGFs is unresolved. It has been suggested that bioavailable circulating IGF-1 concentrations are regulated by IGFBP-1 and IGFBP-2 rather than IGFBP-3, despite the higher peripheral concentrations of IGFBP-3 (Kaaks et al, 2000). A small case–control study found elevated circulating concentrations of both, IGFBP-2 and IGFBP-3, among patients with colorectal cancer (El-Atiq et al, 1994). Mutations in IGFBP-3 have been found in colorectal cancers, although the functional significance of such mutations is not known (Zou et al, 1998).

In conclusion, among Chinese men in Shanghai, circulating IGF-2 and IGFBP-3 levels were elevated in colorectal cancer cases up to 8 years before the clinical diagnosis of cancer. These results are potentially relevant for colorectal cancer screening and colorectal adenoma surveillance.

### ACKNOWLEDGEMENTS

This research was supported by grants R01 CA43092 and R35 CA53890 from the United States National Cancer Institute, Bethesda, Maryland, and grant P30 ES07048 from the United States National Institute of Environmental Health Sciences, Research Triangle Park, North Carolina. The effort of Dr Nicole Probst-Hensch was supported by grant 3233-054996.98 from the Swiss National Science Foundation, Bern, Switzerland. We thank D-L Qing, MS, Y-L Zhang, RN, and J-R Cheng of the Shanghai Cancer Institute for their assistance in data collection and management, the staff at the Shanghai Cancer Registry for their assistance in verifying cancer diagnosis, and Kazuko Arakawa, MS, of the University of Southern California for her assistance in data management and analysis.

*© 2001 Cancer Research Campaign*

### REFERENCES

Cadoret A, Baron-Delage S, Bertrand F, Kornprost M, Groyer A, Gespach C, Capeau J and Cherqui G (1998) Oncogene-induced up- regulation of Caco-2-cell proliferation involves IGF-II gene activation through a protein kinase C-mediated pathway. *Oncogene* **17**: 877–887

Cui H, Horon I, Ohlsson R, Hamilton SR and Feinberg AP (1998) Loss of imprinting in normal tissue of colorectal cancer patients with microsatellite instability. *Nature Med* **4**: 1276–1280

Di Popolo AD, Memoli A, Apicella A, Tuccillo C, Palma A, Ricchi P, Acquaviva AM and Zarrilli R (2000) IGF-II/IGF-I receptor pathway up-regulates COX-2 mRNA expression and PGE2 synthesis in Caco-2 human colon carcinoma cells. *Oncogene* **19**: 5517–5524

EI-Atiq F, Garrouste F, Remacle-Bonnet M, Sastre B and Pommier G (1994) Alterations in serum levels of insulin-like growth factors and insulin-like growth-factor-binding proteins in patients with colorectal cancer. *Int J Cancer* **57**: 491–497

Ferry RJ, Cerri RW and Cohen P (1999) Insulin-like growth factor binding proteins: new proteins. new functions. *Horm Res* **51**: 53–67

Freier S, Weiss O, Eran M, Flyvbjerg A, Dahan R, Nephesh I, Safra T, Shiloni E and Raz I (1999) Expression of the insulin-like growth factors and their receptors in adenocarcinoma of the colon. *Gut* **44**: 704–708

Giovannucci E (1999) Insulin-like growth factor-1 and binding protein-3 and risk of cancer. *Horm Res* **51 (Suppl 3)**: 34–41

Giovannucci E, Pollak MN, Platz EA, Willett WC, Stampfer MJ and Majeed N et al (2000) A prospective study of plasma insulin-like growth factor-1 and binding protein-3 and risk of colorectal neoplasia in women. *Cancer Epidemiol Biomark Prev* **9**: 345–349

Hassan AB and Howell JA (2000) Insulin-like growth factor II supply modifies growth of intestinal adenoma in Apc(Min/+) mice. *Cancer Res* **60**: 1070–1076

Hosmer DW and Lemeshow S (1989) Applied Logistic Regression. John Wiley & Sons: New York

Jones JI and Clemmons DR (1995) Insulin-like growth factors and their binding proteins: biological actions. *Endocrine Reviews* **16**: 3–34

Kaaks R, Toniolo P, Akhmedkhanov A, Lukanova A, Biessy C, Dechaud H, Rinaldi S, Zeleniuch-Jacquotte A, Shore RE and Riboli E (2000) Serum C-peptide insulin-like growth factor-I (IGF-I). IGF-binding proteins and colorectal cancer risk in women. *J Natl Cancer Inst* **92**: 1592–1600

Kansra S, Ewton DZ, Wang J and Friedman E (2000) IGFBP-3 mediates TGF beta 1 response in colon cancer cells. *Int J Cancer* **87**: 373–378

Lamonerie T, Lavialle C, Haddada H and Brison O (1995a) IGF- 2 autocrine stimulation in tumorigenic clones of a human colon-carcinoma cell line. *Int J Cancer* **61**: 587–592

Lamonerie T, Lavialle C, de Galle B, Binoux M and Brison O (1995b) Constitutive or inducible overexpression of the IGF-2 gene in cells of a human colon carcinoma cell line. *Exp Cell Res* **216**: 342–351

Ma J, Pollak MN, Giovannucci E, Chan JM, Tao Y, Hennekens CH and Stampfer MJ (1999) Prospective study of colorectal cancer risk in men and plasma levels of insulin-like growth factor (IGF)-1 and IGF-binding protein-3. *J Nati Cancer Inst* **91**: 620–625

Manousos O, Souglakos J, Bosetti C, Tzonou A, Chatzidakis V, Trichopoulos D, Adami H-O and Mantzoros C (1999) IGF-I and IGF-II in relation to colorectal cancer. *Int J Cancer* **83**: 15–17

Renehan AG, Painter JE, O'Halloran D, Atkin WS, Potten CS, O'Dwyer ST and Shalet SM (2000) Circulating insulin-like growth factor II and colorectal adenomas. *J Clin Endocrinol Metab* **85**: 3402–3408

Szepeshazi K, Schally AV, Groot K, Armatis P, Halmos G, Herbert F, Szende B, Varga JL and Zarandi M (2000) Antagonists of growth hormone-releasing hormone (GH-RH) inhibit IGF-II production and growth of HT-29 human colon cancers. *Br J Cancer* **82**: 1724–1731

Thissen JP, Ketelslegers JM and Underwood LE (1994) Nutritional regulation of the insulin-like growth factors. *Endocrine Reviews* **15**: 80–101

Yuan JM, Ross RK, Wang XL, Gao Y-T, Henderson BE and Yu MC (1996) Morbidity and mortality in relation to cigarette smoking in Shanghai. China. *JAMA* **275**: 1646–1650

Zou T, Fleisher A, Kong D, Yin J, Souza R, Wang S and Smolinski KN (1998) Abraham JM, Meltzer SJ. Sequence alterations of IGFBP3 in neoplastic and normal gastrointestinal tissues. *Cancer Res* **58**: 4802–4804

*© 2001 Cancer Research Campaign*
